# Paris on the Mekong: using the aid effectiveness agenda to support human resources for health in the Lao People's Democratic Republic

**DOI:** 10.1186/1478-4491-7-16

**Published:** 2009-02-25

**Authors:** Rebecca Dodd, Peter S Hill, Dean Shuey, Adélio Fernandes Antunes

**Affiliations:** 1 World Health Organization, Geneva, Switzerland; 2 School of Population Health, The University of Queensland, Herston, Queensland, Australia

## Abstract

**Background:**

This study examines the potential of aid effectiveness to positively influence human resources for health in developing countries, based on research carried out in the Lao People's Democratic Republic (Lao PDR). Efforts to make aid more effective – as articulated in the 2005 Paris Declaration and recently reiterated in the 2008 Accra Agenda for Action – are becoming an increasingly prominent part of the development agenda. A common criticism, though, is that these discussions have limited impact at sector level. Human resources for health are characterized by a rich and complex network of interactions and influences – both across government and the donor community. This complexity provides a good prism through which to assess the potential of the aid effectiveness agenda to support health development and, conversely, possibilities to extend the impact of aid-effectiveness approaches to sector level.

**Methods:**

The research adopted a case study approach using mixed research methods. It draws on a quantitative analysis of human resources for health in the Lao People's Democratic Republic, supplementing this with a documentary and policy analysis. Qualitative methods, including key informant interviews and observation, were also used.

**Results:**

The research revealed a number pathways through which aid effectiveness is promoting an integrated, holistic response to a range of human resources for health challenges, and has identified further opportunities for stronger linkages. The pathways include: (1) efforts to improve governance and accountability, which are often central to the aid effectiveness agenda, and can be used as an entry point for reforming workforce planning and regulation; (2) financial management reforms, typically linked to provision of budget support, that open the way for greater transparency and better management of health monies and, ultimately, higher salaries and revenues for health facilities; (3) commitments to harmonization that can be used to improve coherence of donor support in areas such as salary supplementation, training and health information management.

**Conclusion:**

If these opportunities are to be fully exploited, a number of constraints will need to be overcome: limited awareness of the aid effectiveness agenda beyond a core group in government; a perception that this is a donor-led agenda; and different views among partners as to the optimal pace of aid management reforms. In conclusion, we recommend strategic engagement of health stakeholders in the aid effectiveness agenda as one means of strengthening the health workforce.

## Background

Human resources for health (HRH) are characterized by a rich and complex network of interactions and influences – both across government and the donor community. Workforce planning and recruitment are influenced by public administration systems; salary rates and conditions for health workers intersect with those of the broader civil service; and pre-service vocational training, in-service training and continuing professional development engage stakeholders not only in education, but also in trade and foreign policy. At a higher level, whole-of-government agendas such as poverty reduction, decentralization and privatization also influence the profile, regulation and deployment of the health workforce. This complexity provides a good prism through which to assess the potential of the aid effectiveness agenda to support HRH development. This in turn gives us an insight into the dynamics of the development process and opportunities for aid management reform.

The Paris Declaration on Aid Effectiveness [1] has been endorsed by more than 100 developing and developed countries as well as by key multilateral agencies, the international finance institutions and civil society organizations. It sets out principles to guide donor support built around the three pillars of the aid effectiveness agenda: harmonization and simplification of donor policies and procedures; alignment behind national priorities and use of country systems; and a focus on results as measured in improved development outcomes. Support for the Paris Declaration was recently reiterated at the Third High-Level Forum on Aid Effectiveness, a meeting of development partners and developing countries held in September 2008, though stakeholders also noted a range of challenges to its implementation. Among these were the need to broaden the range of actors in government involved in aid effectiveness processes and to intensify efforts to apply aid effectiveness approaches at sector level [2].

This study examines the potential of aid effectiveness to positively influence HRH in developing countries, based on research carried out in the Lao People's Democratic Republic (hereafter the Lao PDR). The capital of Lao PDR is Vientiane. On the banks of the Mekong River, with its broad boulevards and distinct French colonial heritage, it gives its name to a localized version of the Paris Declaration: the Vientiane Declaration, signed in September 2006 by 23 partner countries and organizations providing aid to the Lao PDR.

According to the Organisation for Economic Co-operation and Development (OECD), donors committed USD 36.7 million to health in the Lao PDR in 2005, and USD 20.8 million in 2006 (Table 1, also Additional File 1). These figures are in line with those published by the Government of the Lao PDR, which recorded disbursements of USD 36.6 million to health in financial year 2005–2006 [3]. OECD lists 173 separate health or population "activities" (in OECD terminology) for Lao covering the period 2001–2006, with a median value of USD 0.23 million. In general, an "activity" signifies allocation of funds to a specific project or programme. However, donors sometimes choose to report at a more detailed level, in which case a "reported activity" may represent a component of a project. But there are also cases where activities are aggregated, so a single "reported activity" can be the sum of several activities.

**Table 1:** Health aid commitments to the Lao PDR (USD, millions) (See Additional File 1)

	**2001**	**2002**	**2003**	**2004**	**2005**	**2006**	**Total**
Australia	13.67	0.19	0.08	1.53	0.59	0.19	16.25

Belgium	0.84	0.77	0.77	0.43	1.18	1.43	5.42

Canada		4.31		0.50		0.04	4.85

France	0.35	0.59	1.25	1.49	1.46	1.90	7.04

Germany		1.27			0.50	0.57	2.34

Ireland						0.11	0.11

Italy				0.04			0.04

Japan		2.66	5.31	9.82	5.42	10.10	33.31

Luxembourg	0.48		9.17	4.19	3.58	2.20	19.63

New Zealand				0.01		0.48	0.49

Norway		0.10		0.06	0.05		0.21

Sweden	1.92				0.00	1.08	3.00

United Kingdom				0.56			0.56

United States		2.03	2.11	0.68	0.00		4.82

EC	2.23		0.97				3.20

GFATM			19.67		7.48		27.15

IDA					15.00	1.13	16.13

UNAIDS	0.23	0.24	0.17		0.28		0.93

UNFPA	2.48	2.28	1.47				6.22

UNICEF	1.20	0.94	0.79	0.97	1.10	1.52	6.52

Total	23.40	15.39	41.76	20.26	36.65	20.77	158.23

Source: Creditor Reporter System, OECD/DAC

The largest entry for this period was a USD 15.9 million grant from the Global Fund to Fight Aids, Tuberculosis and Malaria (GFATM) for infectious disease control, but the majority were for much smaller amounts, with 134 of the 173 activities having a value of less than USD 1 million dollars. This suggests a high degree of fragmentation in donor support, and quite high transaction costs for government in managing many separate activities. Activities are classified broadly – for example, as "basic health care" or "reproductive health care", thus it is not possible to disaggregate specific amounts spent on human resources for health.

This level of donor support is low in comparison to many other low-income countries [4], but it is still three times higher than government spending. Per capita health expenditure was estimated at USD 22 per capita in 2006, of which 75% comes from households [5]; of public expenditure, between 70% and 75% is financed by donors, the remainder by government [6]. Further, the landscape of health donors is complicated: Japan, Luxembourg and the GFATM are the major contributors, but there are 12 other bilateral donors active in health as well as the European Commission, World Bank, Asian Development Bank and various United Nations agencies. This points both to the importance and influence of external support in the sector and to the synergies offered by the aid effectiveness agenda in making optimal use of limited resources.

## Methods

This research adopted a case study approach using mixed research methods. It drew on a quantitative analysis of HRH in the Lao PDR undertaken by the World Health Organization (WHO) and the Ministry of Health (MOH) [7], supplementing this with a documentary and policy analysis, examination of the academic literature, government and donor agency policy, reports and publications, unpublished research and reviews. Qualitative methods, including key informant interviews and observation, focused on the potential linkages between HRH and the aid effectiveness agenda.

A total of 23 key-informant interviews were conducted. Stratified selection was used to ensure a balance of informants across ministries of health and finance, the Public Administration and Civil Service Authority (PACSA) and development partners. All major partners active in health and human resources development were interviewed: the Asian Development Bank, European Commission, France, Japan, Luxembourg, the Joint United Nations Programme on HIV/AIDS (UNAIDS), the United Nations Development Programme (UNDP), the United Nations Population Fund (UNFPA), the United Nations Children's Programme (UNICEF), WHO and the World Bank, as well as major non-governmental organizations (NGOs). Information on the GFATM activities was collected from its web site, and via those partners active in the Country Coordinating Mechanism, which oversees GFATM activities.

The question guide used during the interviews was developed prior to the field component, and reviewed by colleagues working on HRH in WHO Geneva. Two interviewers (RD and PSH) attended each interview, alternating roles as lead interviewer and note-taker.

Notes from interviews were transcribed within 12 hours and their accuracy and comprehensiveness corroborated by both interviewers. Findings were triangulated across different interviewees, and a preliminary presentation of the key findings made to the WHO country office to test the initial analysis. An internal peer review process within WHO was also carried out.

### An overview of HRH challenges in the Lao PDR

The workforce analysis undertaken by WHO and the MOH [7] highlights a range of HRH challenges in the Lao PDR. These include inadequate training, low salaries and inadequate non-monetary incentives, all of which have led to a geographical maldistribution of health workers and poor productivity. Skilled professionals are concentrated in the capital and economically better-off regions and there are corresponding gaps at the periphery. This situation is typical of many low-income countries and is not specific to the Lao PDR [8,9].

**Figure 1:**
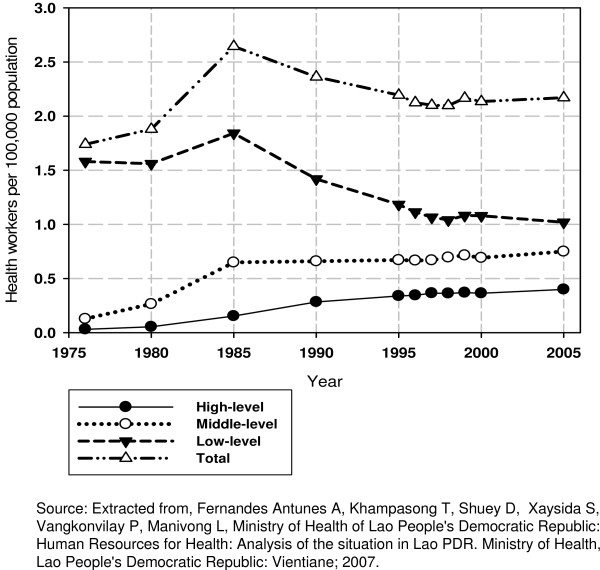
**Evolution of density for the three main types of health worker (low-, mid- and high-level staff) from 1976 until 2005**.

Table 2 and Fig. 1 show that while the ratio of health workers to population has grown steadily over the last three decades, the most senior category of the profession (mainly physicians) has grown most. Medical-to-nursing ratios fell from 1:9.9 in 1976 to 1:3.7 in 1995, with 2005 figures showing only 1.8 nurses per medical staff (physicians and medical assistants) [7]. Physicians-to-nursing/medical assistants rations also fell over time from 1:54.8 in 1976 to 1:5.4 in 1995, reaching 4.5 nurses/medical-assistants per medical graduate in 2005 [7]. This structure has been purposefully established over time, as a government decision that saw high-level medical education as the preferred solution to inadequate health coverage [10].

**Table 2:** Evolution of health worker density per 100 000 inhabitants from 1976 to 2005

**Professional level**	**Years of training**	**1976**	**1980**	**1985**	**1990**	**1995**	**2000**	**2005**
Postgraduate/High-level*	<4	0.03	0.05	0.15	0.28	0.34	0.36	0.40

Mid-level*	2 to 3	0.13	0.26	0.65	0.66	0.67	0.69	0.75

Low-level *	<2	1.58	1.56	1.84	1.42	1.18	1.08	1.02

Total		1.74	1.88	2.64	2.36	2.19	2.13	2.17

*Classification reflects that used by Ministry of Health in the Lao PDR

Source: Extracted from, Fernandes Antunes A, Khampasong T, Shuey D, Xaysida S, Vangkonvilay P, Manivong L, Ministry of Health of Lao People's Democratic Republic MOH: Human Resources for Health: Analysis of the situation in Lao PDR. Ministry of Health, Lao People's Democratic Republic: Vientiane; 2007.

Recent data on intake numbers for medical training and appointment quotas for different cadres at provincial level continue to reflect historical patterns. In 2005, there were 4163 students enrolled in medical training, of whom 28% were "high-level" (and 14% physicians), 63% were "mid-level" (and 41% nurses), and just 8% were "low-level" or primary-care workers. In terms of allocation, in 2005 physicians accounted for 48 of 441 (11%) of health staff allocated across the Lao PDR. There is also a strong bias in favour of the centre in the allocation of new staff, with 39% of new recruits being sent to Vientiane in 2005, including 28 of the 48 newly-qualified doctors. By contrast, most of the senior posts in rural and poor regions remain unfilled, forcing local authorities to rely on low-level staff.

**Figure 2:**
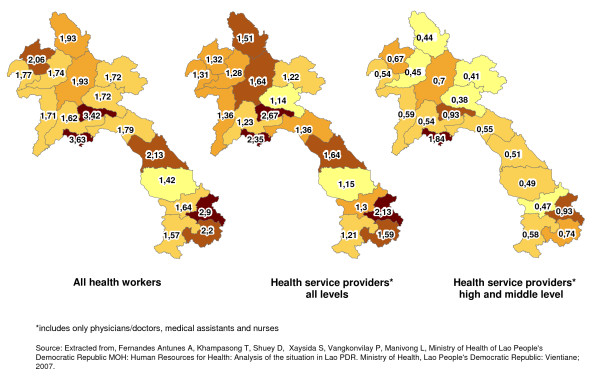
**Maps showing the ratios of the different health worker categories per 1000 inhabitants per province**.

Overall, health workers are disproportionately concentrated in the capital: Vientiane has 3.63 health workers per 1000 inhabitants (Fig. 2). Of the remaining 17 provinces, 15 have a health worker density of less than 2.5 health workers per 1000, and in the more remote, southern provinces density drops to 1.4 per 1000. This distortion is even more pronounced when it comes to high-level and mid-level health service providers (physicians, medical assistants and nurses), with 1.84 such health workers per 1000 in the capital and all other provinces recording rates of less than one health worker per 1000 people [7].

Low salaries (discussed further below) are one important reason that health workers have a strong preference for urban areas, where they have opportunities to earn supplementary income from private practice. In the Lao PDR as elsewhere, educational and career-development opportunities, better schools and health care for families attract and retain staff in cities.

Midwifery skills are a conspicuous gap in the health workforce [11]. While nursing graduates are expected to have competence in both nursing and midwifery, graduates typically have very limited clinical obstetric experience, as very few births take place in public facilities. Only 103 midwives and 63 auxiliary midwives currently work in clinical obstetric roles [11]. With maternal mortality in the Lao PDR estimated at 405 per thousand live births [12] and only 19% of deliveries assisted by skilled birth personnel, establishing a cadre of health workers with midwifery skills is a recognized priority for government and donors alike. The low skills of nursing graduates also points to the broader issue of inadequate standards in training, which affects all cadres of the workforce.

## Results and discussion

In this section we explore the intersections between HRH and aid effectiveness in the Lao PDR in relation to four specific issues: workforce planning; training; salaries and supplements; and financial management. In each case we present current challenges, map existing examples of how aid effectiveness is being used to address those challenges and discuss opportunities for further action. We argue that one of the most promising aspects of the aid effectiveness agenda is its broad scope and complex network of cross-governmental links, which provides a mechanism for mediating across the web of stakeholders and interest groups that characterize HRH. A number of concerns are also presented.

### Workforce planning

As discussed above, the current workforce profile in the Lao PDR presents challenges in relation to the distribution of health staff and the balance between health cadres – with distortions towards the centre and a relative undersupply of primary care workers: "Many hospitals do not have enough nurses, which creates problems for basic patient care" (said a senior MOH staff member).

The quota system that allocates staff to provinces locks-in the historical dominance of doctors over nurses. Each year provinces and programmes submit a request for new posts, based on exits (deaths, transfers, retirements) and estimated needs. These requests are compiled by the Ministry of Health and then forwarded to the PACSA for consideration. Informants suggest that because requests from the provinces are so disproportionate to supply and because PACSA does not have the necessary technical perspective to discriminate between competing staffing needs in an environment of resource constraints, historical patterns tend to be maintained. According to a staff member of a multilateral agency: "PACSA does not have an overview of staffing allocation or technical awareness... but it is open to more rational case presentations".

Two aid effectiveness initiatives are affecting this challenge. First, within the health sector, structures for dialogue have been established that allow a shared analysis of inefficiencies in the quota system. Based on two important reports on the health workforce – by WHO and the MOH in June 2007 and by UNFPA in 2008 – the Sector Working Group on health is forging a common understanding on this issue between donors and with government. The Sector Working Group is chaired by the MOH and co-chaired by WHO and Japan. It meets twice a year at ministerial and ambassadorial level to discuss overall policy directions in the sector, and four times a year at the operational level (deputy-directors of MOH departments and health advisers from the development partners) to focus on operational coordination. Three subgroups have also been established: on financing, human resources and maternal and child health.

Second, looking across sectors, efforts to strengthen capacity for planning are being implemented under the auspices of the Vientiane Declaration Action Plan. Capacity development frameworks have been developed for key sectors (transport, education and health) and in relation to cross-cutting issues (aid effectiveness and emergency preparedness). These will build managerial skills – e.g. for planning and budgeting – within the central government and in selected line ministries. Of specific relevance to health workforce planning is the development of a database on human resources management by PACSA, with United Nations support. This database was in turn adapted by the Ministry of Health, and used to register all health workers in five provinces, providing information on workforce capacities and gaps to be used in planning.

This combination of sectoral and cross-sectoral aid effectiveness initiatives establishes the necessary structures and capacities to strengthen planning and governance functions in the ministry of health and in central government, and opens the door to reform the quota system. This in turn paves the way for a more rational, integrated and needs-based approach to workforce planning in health.

### Training

Challenges in relation to training of the health workforce include the lack of intersection between pre-service vocational and in-service training, poor coordination between partners providing support for training, and the need to increase the level of clinical experience offered to medical students.

One of the central findings of the research, triangulated across a range of respondents, is that donor support for both pre-service and in-service training has been non-harmonized and supply-led, with government reluctant to take a lead role in coordination. For example, the lack of coordination between partners supporting specialist training is such that they cannot agree what language should be used to deliver classes. Offers of support often come directly from developed-country hospitals direct to the MOH, and remain *ad hoc *rather than integrated into a comprehensive system of postgraduate training. "Coordination of in-service and short training between vertical programmes is [also] an issue. We would like these to be integrated, but don't know how", said a senior MOH staff member.

Poor coordination in support for training has been exacerbated by a stop-start approach to training of new cadres: training for medical assistants was supported, then discontinued and may yet start again; training for midwives has followed the same pattern [11]. Primary Health Care workers were created as a low-level cadre, but are now being upgraded to medical assistants [7]. Donors were often involved in the decision to make changes in cadres, although government took the final decision.

With its emphasis on harmonization, the aid effectiveness agenda has catalysed a number of initiatives to address the coordination challenge. First, a strategic plan on HRH development is in the making, which will provide a framework behind which donors can in future align their support. Second, some partners are already aligning their support for *primary *health care training with the MOH's *Healthy Villages *scheme. Third, France is leading an effort to coordinate offers of support to medical specialist training. Key to the success of these initiatives will be the close and continued involvement of government.

On curriculum revision, the Sector Working Group subgroup on HRH provides a mechanism to address the relative neglect of training for nursing, midwifery and allied professions and to provide a greater emphasis on clinical experience. "In practice, training is theoretical, not competence-based", commented one bilateral partner. The lack of good practical training sites is a key issue in this regard. The research revealed a common concern among development partners supporting the sector on these points and a shared view that the HRH subgroup was the best forum for formulating a response. Upgrading the capacity of health workers appears as the third strategic programme area in the health chapter of the Lao PDR's Sixth Socio Economic Development Plan [15], providing a further impetus for donors to take a more coherent approach to this issue.

Simultaneously, the ASEAN Free Trade Agreement (AFTA) is driving a review of health training curricula. Health partners and government could use this review as an entry point to pursue reforms, including improving clinical competences. Under AFTA, free movement of nurses is allowable once national training curricula meet certain quality criteria. A process to determine equivalence among doctors is under negotiation, which is also driving reform of their curriculum. This reframes health professional training in terms of regional development and provides a political impetus, beyond the MOH, to raise curriculum standards. But there are also concerns. MOH interviewees are worried that the public system will suffer a brain drain of health professionals if foreign-managed facilities are established, as AFTA allows.

This points to the need for coherence across different aspects of development policy: an area where the aid effectiveness agenda has the potential to deliver, but has yet to do so. Though the drivers of AFTA are primarily economic, the agreement has clear implications for health worker development, not only in the Lao PDR but also across South-East Asia. Development partners interested in setting comparable quality standards across the region will need to ensure coherence between their support for economic development (including trade liberalization) and support for health. By taking a holistic view of the development agenda, aid effectiveness discussions provide a forum where this synergy could be achieved.

### Salaries and supplements

There is universal agreement among development partners in the Lao PDR that the salaries of health workers are unacceptably low, reported by MOH to be just USD 50 per month in remote areas. Low salaries translate into low productivity, with patient contacts in the Lao PDR standing at roughly 7% of international averages [16].

Even so, opportunities for increasing base pay levels are limited. Some 80% of the domestically-financed health budget is already spent on salaries [7], which means that available fiscal space for further increases is very limited. Moreover, increasing salaries for health workers independently from other civil service staff was regarded by informants as inappropriate: "We need a national solution – not one just for MOH", suggests one donor partner. The Ministry of Finance's view is that low salaries are not the most pressing problem facing the sector: "Is the problem really salaries, or the system as a whole? I'm not convinced that raising salaries will improve health. The whole system needs reform." Given these views and the fact that a 20% increase in civil service pay was awarded in October 2007, further government-funded increases in health worker pay are unlikely in the near future.

This begs the question: Could salary increases be funded by donors? Most donor support for health is classified as capital expenditure in the national budget (see Table 3, also Additional File 2), but in practice much of this is spent on things that would normally be classified as recurrent costs, such as salary supplements. However, the potential to fund base salary increases through donor support appears to be limited. The authors encountered a resistance to this idea within the Ministry of Finance: "Paying salaries is the business of government," said one senior staff member.

**Table 3:** Total public and public health expenditure, 2002–2005 (as % of GDP) (See Additional File 2)

	**2002**	**2003**	**2004**	**2005**
Total expenditure (including debt)	19.6	19.5	15.7	19.9

Health expenditure as % of total public	0.98	1.13	0.68	1.14

• Domestic expenditure	0.49	0.34	0.28	0.31

• Foreign financed	0.49	0.79	0.4	0.83

Structure of expenditure				

• Capital expenditure, in total	0.65	0.9	0.43	0.87

• Domestically financed	0.16	0.1	0.04	0.04

• Foreign financed	0.49	0.79	0.4	0.83

• Recurrent expenditure, total	0.33	0.23	0.25	0.27

• Salaries/Wages	0.21	0.16	0.19	0.17

• Operations and Maintenance	0.11	0.07	0.06	0.1

Further, donors argue that government needs to first demonstrate its commitment to increasing salaries. As overall government spending on health appears to be decreasing – dropping from 6.4% to 3.2% of the total budget between 2004–2005 and 2005–2006 [17], the chances of this are not high. Projected revenues from a planned hydroelectric scheme are one promising source of funds for salary increases [18] – the Nam Theun 2 dam should generate USD 20 billion over its lifetime. While there is a commitment to spending a good share of this revenue on the social sectors, the exact amounts and modalities are not yet clear.

Through its focus on human development, poverty reduction and the MDGs the Vientiane Declaration provides donors with the mandate needed to advocate pay increases in the social sector. The opening statement of the Vientiane Declaration is as follows:

We, the Government of the Lao People's Democratic Republic and the Partners in Development, seek to take appropriate monitorable actions to make aid more effective and assist the country in achieving the Millennium Development Goals (MDGs) by 2015 and the long-term development goal of exiting the status of least-developed country by 2020.

The mechanisms of aid effectiveness – coordination groups, etc – provide the means through which this advocacy can be carried out.

A convincing analysis of how new monies could be used to fund pay increases in the health sector is now needed; the MOH and WHO have already done some work in this area [7]. Sector and inter-sector coordination mechanisms could be used to develop common positions, mobilize support and overcome the political obstacles outlined above. A coherent position within donor agencies – between their poverty reduction and health teams – is essential.

In an environment where base salary levels and productivity are low, the indirect incentives and allowances provided by donors to health staff implementing their projects become very important. These include travel and meeting allowances, access to transport and computers, and so on. Interviewees report that there is no standardization of incentives between partners or with the MOH, with some partners paying supplements that are much higher than others. Further, the payment of travel allowances is creating perverse incentives in terms of service delivery. Lack of transparency on this issue made it difficult for the research team to gather firm evidence, but anecdotal reports suggest that problems are significant: "Nurses working in HIV wards earn USD 100 a month more than general nurses, working in the adjacent ward." "Staff prefer to do outreach rather than facility-based immunization in order to get the overnight per diem," reports United Nations staff. In some cases, supplementation practices ran counter to donor policy, but were pursued nonetheless.

The Sector Working Group is trying to tackle this issue, but progress is slow. An attempt to standardize rates across the United Nations and some partners fell apart when others failed to join. There are also differing views within the Group on how proactive donors should be in pushing this issue with government. "Every sector needs to find its own way – not necessarily follow other countries," said one bilateral partner, adding that, according to the principle of ownership, partners should be more patient in waiting for government to take the lead in coordination. Others disagree, feeling that a more open and candid dialogue is needed within the Group: "Difficult issues simply aren't discussed," said one multilateral partner.

Still others feel that, given the incentives associated with current ways of doing business, government is unlikely to initiate change: "The direct project allowances of top managers in the MOH and their income from indirect revenues such as per diems creates a strong incentive to maintain the status quo" said a United Nations informant.

This tension – of differing expectations and approaches within the donor community – is an emerging theme in reports monitoring the implementation of the Paris Declaration, and is thus not unique to the Lao PDR [19].

### Financial management

Budget support is the process by which donors deliver their financial assistance directly into the government budget and it is mixed with domestic revenues [20]. It is seen by some as one of the more effective forms of aid because it avoids many of the costs and inefficiencies associated with multiple projects, it is easier to align with recipient priorities and it opens the way to a broader, strategic dialogue on economy-wide issues [21]. The counter argument is that if accountability and governance are poor – as they often are in developing countries -resources may well be misspent.

Whatever its pros and cons, budget support has become increasingly associated with effective aid [22]. In the Lao PDR as in many other countries, budget support is linked to efforts to improve the public financial management systems through which money is channelled. This, in turn, can have a positive impact on the health sector and HRH, as discussed below.

Currently, budget support in the Lao PDR is delivered through the World Bank's Poverty Reduction Support Operation (PRSO), which is financed by the World Bank, the European Commission and Japan. In 2008, the PRSO was worth USD 20 million, equivalent to just under 10% of the overall government budget. The European Commission also links additional support of EUR 1 million per annum, 2009–2011, to progress towards certain conditions related to the PRSO, including finalization of a health sector financing strategy. While details of this strategy are not yet defined, it is expected to improve planning, management and monitoring of health resources through links to a Medium Term Expenditure Framework that would provide a common planning and monitoring framework for government and donor resources.

A second example of how aid effectiveness reforms positively influence financial planning in health is a proposed new budget law, supported by World Bank as part of public financial management reform, which would ensure that central government received a share of the revenues collected at provincial level. The bulk of domestic revenues is currently raised and spent at provincial level [23]. This means that each province's capacity to provide health services is contingent on its own revenue-raising potential, and that the centre has very little leverage to regulate. This, combined with the financial and administrative autonomy of facilities, has created a situation in which fees charged by health providers differ from province to province and there is no standardized approach to regulation of private practitioners. The public financial management reforms associated with budget support have the potential to strengthen the centre, and in so doing to improve opportunities for regulation.

Despite these synergies, the authors encountered limited knowledge and commitment to aid effectiveness beyond "upstream" ministries such as planning and finance. The agenda has yet to engage line ministries such as health, lower levels of government, or nongovernmental partners. Further, there is a widely-held view that the Vientiane Declaration is a donor product that does not yet have the full support of government. "The Vientiane Declaration has been pushed primarily by donors," said one United Nations staff member. (Literature on Sector Wide Approaches notes that partners, not government, are often at the forefront of coordination efforts [13,14].)

Respondents offered a range of likely reasons for this. First, the potential of aid effectiveness to deliver improvements in development outcomes is not always immediately apparent. "The current debate is too broad and superficial," said one bilateral partner. Second, the transaction costs associated with coordination are often high – as demonstrated by difficulties encountered by the Sector Working Group in standardizing incentives and allowances. "The benefits for the MOH are unclear," said a United Nations staff member. Third, there are substantial incentives associated with current ways of delivering aid that are difficult to overcome.

## Conclusion

In this article we have focused on what aid effectiveness can do for HRH, demonstrating how this policy instrument is promoting an integrated, holistic response to a range of complex challenges. Some of these challenges are themselves the result of ineffective donor behaviour. Others are rooted in the multisectoral nature of HRH issues. In both cases, the aid effectiveness agenda offers solutions. Examples from the Lao PDR that may provide lessons for other countries include the following.

• Efforts to improve governance and accountability, which are often central to the aid effectiveness agenda, can be used as an entry point for reforming workforce planning and regulation.

• Financial management reforms, typically linked to provision of budget support, open the way for greater transparency and better management of health monies, which in turn have the potential to deliver more resources to the health sector.

• Aid effectiveness' emphasis on harmonization can be used to improve coherence of donor support in areas such as salary supplementation and training.

• The expressed desire for alignment with government policy provides an incentive for the government to develop policies – include HRH plans – that donors can support.

But the pathways of influence are neither simple nor direct. This review has highlighted the difficulties that emerge when aid effectiveness approaches are applied to a specific component of the health system – human resources for health. Two issues emerge, which are also reflected in the global literature. The first is that while aid effectiveness has a conceptual and rhetorical appeal, when operational details are added the consensus may break down, particularly if the status quo is challenged or in-country working methods disrupted. The challenge that the Lao PDR has faced in standardizing salary supplements exemplifies that "the devil is in the details".

The second issue is that aid effectiveness principles are most likely to be operationalized when linked to a substantive reform agenda. The links between budget support and public financial management reform provides a tangible illustration. This is also a lesson that emerges from the literature: SWAps are often a driving force behind health sector reform. In the Lao PDR, human resources development could provide such a framework. The research identified multiple points of intervention in the critical pathways for HRH development where the potential contribution of the aid effectiveness agenda is significant.

Lending confidence to this analysis is the early evidence provided that, in some areas, positive synergies are already emerging and aid effectiveness is already contributing to the resolution of more complex, cross-cutting HRH issues that are difficult to solve from a health sector perspective alone. To optimize the yield from this potential, health sector decision-makers will need to actively engage their counterparts working on aid effectiveness and overcome identified challenges. Conversely, these counterparts will need to be open to collaboration at sector level. In our view, this is an effort worth making, with the potential to deliver benefits to both sides.

## Competing interests

The authors declare that they have no competing interests.

## Authors' contributions

RD conceived the project, developed the research design, undertook the key informant interviews, prepared the analysis and drafted the manuscript; PSH assisted with the research design, undertook the key informant interviews, collaborated in the analysis and offered critical comments in the drafting and review of the manuscript; AFA and DS undertook the quantitative human resources analysis that informed the study and offered critical comments in the drafting and review of the manuscript. All authors have read and approved the final manuscript.

## Supplementary Material

Additional File 1 **Health aid commitments to Lao PDR (USD, millions).** Click here for file

Additional File 2 **Total public and public health expenditure, 2002–2005 (as % of GDP)**. Click here for file
